# A developmental analysis of dimensions of empathy during early adolescence: Behavioral empathy but not cognitive empathy is associated with lower psychopathology

**DOI:** 10.1371/journal.pgph.0001231

**Published:** 2022-11-07

**Authors:** Megan Cherewick, Sarah Schmiege, Emily Hipp, Jenn Leiferman, Prosper Njau, Ronald E. Dahl

**Affiliations:** 1 Department of Community & Behavioral Health, Colorado School of Public Health, Aurora, CO, United States of America; 2 Department of Biostatistics & Informatics, Colorado School of Public Health, Aurora, CO, United States of America; 3 Health for a Prosperous Nation, Dar es Salaam, Tanzania; 4 Institute of Human Development, University of California Berkeley, Berkeley, CA, United States of America; Transcultural Psychosocial Organization Nepal / KIST Medical College, NEPAL

## Abstract

Construct definitions of empathy have sought to distinguish between different dimensions of empathetic capacity that are significantly associated with psychological distress or wellbeing. Research has provided substantial evidence differentiating affective and cognitive empathy; however, more recent research has cited the importance of a third domain represented by empathetic behaviors and compassionate intent to comfort others. Examining developmental and maturational stage during the rapid transitional period of early adolescence is needed to model developmental trajectories, mechanisms of change and mental health outcomes. This study aims to assess relationships between pubertal developmental stage, dimensions of empathy, and depression, anxiety and externalizing behaviors among early adolescents. A cross-sectional survey among young adolescents ages 9–12 years was conducted in Dar es Salaam, Tanzania. The relationships between pubertal developmental stage, affective, cognitive, and behavioral empathy scores and internalizing and externalizing symptoms were evaluated using hierarchical regression modeling. Structural equation modeling was used to test a theoretical model of structural paths between these variables. A sample of 579 very young adolescents (270 boys and 309 girls) aged 9–12 years participated in the study. Pubertal development scale scores were associated with affective, cognitive, and behavioral empathy. Adolescents who had greater behavioral empathy scores reported lower internalizing and externalizing symptoms. Adolescents who had transitioned further through puberty and had higher cognitive empathy scores and reported higher internalizing symptoms. These findings support the importance of pubertal developmental stage in assessing risk and protective paths to mental health during adolescence. While empathetic capacity is widely perceived to be a positive trait, dimensional analysis of empathy among early adolescents indicates that behavioral skills and compassionate acts may be particularly protective and promote positive mental health outcomes.

## Introduction

Empathy has been defined as a character trait representing the affective and moral capacity to understand emotional states of others [1]. Researchers have also sought to distinguish between sympathy, empathy, and compassion and more recent research has sought to differentiate dimensions of empathy. A review of empathy measures by Sesso et al., 2021, found that most empathy measures have been shown to be multi-dimensional with affective and cognitive subdimensions most often cited in validation studies [2]. Sympathy has been equated with affective empathy, an emotionally reactive response from the individual observing distress, while cognitive empathy is the capacity to intellectually understand another person’s experience, appraise and identify with emotional states in ways that goes beyond the threshold of affective empathy or sympathy [3–6].

As a latent construct, empathy has been shown to be significantly associated with prosocial behavior [7] and externalizing problems, such as aggression and substance misuse [8, 9]. Studies exploring dimensions of empathy and relationships with psychopathology have found that cognitive empathy predicts higher friendship quality [10–12]. More recently, studies of empathy have considered a third dimension of empathy in addition to affective and cognitive empathy. This dimension, ‘intention to comfort’ represents compassionate actions and differentiates from both affective and cognitive empathy to include behavioral empathy, the conscious decision making to motivate practice of a prosocial action [13]. This third dimension of behavioral empathy is important because cognitive empathy alone, without the skills to alleviate distress may correlate with co-rumination, the tendency to frequently discuss problems with peers that has been identified as a risk factor for depression during adolescence [14]. Compassionate behavior can be practiced externally in response to another person’s distress or internally to alleviate one’s own distress. Self-compassion has gained increasing attention as a protective asset that can function at the intrapersonal level [15]. In other studies, empathy explained sample variance in mental health outcomes after adjustment for coping struggles and social support [16]. Similarly, research has also recognized that individuals vary in their capacity for *positive empathy*, the capacity to share joy and happiness experienced by others [17].

Empathy research is motivated by growing evidence that empathy is associated with both mental health and wellbeing outcomes [7, 8, 18]. In adolescent research, capacity for empathy has been considered integral to social and moral development [2]. Higher levels of empathy have been associated with prosocial behaviors, higher levels of emotional regulation, and lower aggressive behaviors [19–21]. Evidence suggests the important role empathy plays in other kinds of socially undesirable behaviors such as violence, bullying, and sexual assault perpetration [22, 23]. Research has found that lower empathy is associated with attention deficit and hyperactivity disorder (ADHD) and eating disorders [24]. As a protective asset, affective empathy is associated with emotional regulation in social situations and cooperation towards collective goals [25].

The developmental science of adolescence seeks to enhance precision with which we understand the dynamic maturational period from the onset of puberty to adulthood. Much research to date has focused on differentiating how dimensional factors that comprise empathy explain risk for psychiatric disorders including autism spectrum disorder, ADHD, callous unemotional traits, and aggressive behaviors [26–30]. Developmental neuroscience suggests that dimensions of empathy are explained by different brain systems [31]. For example, development of prefrontal neural circuitry may facilitate empathic responses through higher working memory required to appraise others affective states [32]. Appraisal of other’s distress is tied to several other parts of the brain that are activated during appraisal [31].

Differential associations by gender have important implications for understanding the dimensional structure of empathy. Research indicates that girls have shown higher overall empathetic capacity than boys [33, 34]. In one study, as girls increased in age affective and cognitive empathy increased, whereas in boys, affective, cognitive, and behavioral empathy were negatively associated with age [13]. Interaction effects between developmental age and gender on empathy are plausible and important because girls advance through the pubertal transition at an earlier age than boys on average [35].

Understanding the complex, dynamic, developmental trajectories of the adolescent brain allows a more complete understanding of how to match motivational proclivities and sensitive periods for learning that can shape health trajectories in the near- and long-term. Currently, little is known about high impact developmental periods to promote empathetic capacity. Understanding the significance of empathy during different maturational periods of mental health can inform practice and policy with greater precision and effect.

### Study objectives

The primary objective of this study was to evaluate theoretical relationships between pubertal development stage, mediated by dimensions of empathy (affective, cognitive, and behavioral) on internalizing symptoms (e.g., depression and anxiety) and externalizing symptoms (e.g., aggression and substance use) during early adolescence.

## Materials and methods

### Ethics statement

This study was approved by the University of California Berkeley Committee for Protection of Human Subjects Institutional Review Board (IRB)—(CPHS Protocol Number: 2018-01-10628); in June 2018. The primary local partner in Tanzania, Health for a Prosperous Nation, obtained ethical clearance for these research activities from the National Institute of Medical Research–the local IRB in Tanzania (Ref. NIMR/HQ/R.8a/Vol. IX/2851) in August 2018. All parents/caregivers provided written consent and all adolescents provided verbal assent prior to administration of the survey questionnaire. Due to limited literacy of adolescents, verbal assent was provided and is typical procedural practice in research studies including children in this context.

### Study procedures and sample selection

Participants in this study were recruited from the peri-urban Temeke Municipality in Dar es Salaam, Tanzania to participate in a three-arm comparative effectiveness trial Discover, an intervention to support social emotional mindsets and skills among very young adolescents. A detailed study protocol of the parent study, Discover Learning, is provided elsewhere, including additional details on the subsequent three-arm comparative effectiveness trial and participant recruitment and eligibility criteria [36]. This trial explored whether experiential, social emotional learning for early adolescents ages 10–12, affected mental health and wellbeing outcomes. Data were collected at baseline prior to the start of the intervention in June-July 2019, and presents a cross-sectional view of associations between empathy, pubertal development stage, and internalizing/externalizing symptoms prior to randomization. The analytic sample for this study was comprised of 579 adolescents (270 boys and 309 girls) ages 9–12 (mean age = 10.48; *SD* = 0.55).

### Survey measures

All participants completed a survey at baseline including measures of demographic characteristics (e.g., pubertal development, gender, age), social emotional skills and mindsets (i.e., affective, cognitive and behavioral empathy) and psychosocial assessment (i.e., depression, anxiety and externalizing behaviors). Each measurement scale was selected based on previous use in low- and middle-income countries with adolescent populations. Age and sex were included as covariates in this study.

### Pubertal Development Scale (PDS)

Self-reported pubertal development was measured using the Pubertal Development Scale (PDS) [37], a scale that has been validated and correlated with Tanner staging methods [38]. The PDS has a reported alpha of 0.67–0.70 and includes different questions for boys and girls such as, "Have you noticed a deepening of your voice?” and “Have you begun to grow hair on your face?" for boys; “Have you noticed that your breasts have begun to grow?” and “Have you begun to menstruate?" for girls [37]. Participants responded to three items about perceived changes in maturation on a dichotomous scale. Average ratings were computed so that higher scores indicated higher pubertal development.

### Empathy

Empathy was measured with the Empathy Questionnaire for Children and Adolescents (EmQue-CA) scale [13]. This scale has been validated with Tanzanian adolescents to assess affective empathy, cognitive empathy, and intention to comfort [39]. Affective empathy measures the extent to which adolescents feel the emotional state of the suffering person and includes five items. Cognitive empathy measures the extent to which the adolescent understands why another person is in distress and includes three items. Intention to comfort includes five items that measure the extent to which the adolescent is inclined to actively help or support the suffering person [13]. We refer to the intention to comfort factor as *behavioral* empathy in this study, as it captures a unique third dimension of empathy that highlights not just an understanding of another person’s distress, but how a person intends to behave and react based on that understanding. Response categories asked participants to rate on a 3-point scale whether the statement was 1 = “Not true”, 2 = “Somewhat true” or 3 = “True”. Mean scores were calculated for each subscale such that higher scores reflect higher empathy in that domain. Previous validation of this scale demonstrated adequate internal consistency for cognitive empathy (*α* = 0.89), affective empathy (*α* = 0.65), and behavioral empathy *(α* = 0.75) [39].

### African Youth Psychological Assessment (AYPA)

The African Youth Psychological Assessment (AYPA), measures internalizing and externalizing symptoms in adolescents and has been used previously in Tanzania with minor adaptations [40, 41]. A study to validate the AYPA using a comparison of parent and self-reported ratings on presence of local syndrome terms have been published previously [40]. Among the 166 youth in the validity study, the AYPA demonstrated satisfactory internal reliability, ranging from *α* = 0.70–0.87 for each subscale and further validated subdimensions of depression and anxiety within the internalizing symptom item measures [42]. Table 1 summarizes the items and subscales in this study’s adaptation of the AYPA. Participants were asked each item of the AYPA on a 4-point scale of 1 = “Never”, 2 = “Somewhat”, 3 = “Often”, 4 = “All the time”. The Cronbach’s *α* for this scale lies between 0.72 and 0.88 [40]. All sub-scales had satisfactory to excellent alpha values in this sample, listed as follows: prosocial/adaptive (*α* = 0.72), somatic complaints without medical cause (*α* = 0.74), externalizing problems (*α* = 0.83), and internalizing problems (*α* = 0.88) [40].

**Table 1:** Sample characteristics (*N* = 579).

	*N*	%
Gender		
Boys	270	46.6
Girls	309	53.4
Age		
9	13	2.3
10	276	47.7
11	288	49.7
12	2	0.4
Mean Age (*SD*)	10.48 (0.55)	
Grade		
3	172	29.7
4	261	45.1
5	146	25.2
Live with both parents		
No	173	33.4
Yes	345	66.6
Household profile	Mean	*SD*
Household Size	5.7	2.6
Tanzanian Poverty Score (0–71)^1^	61.4	11.6

Note.

^1^Summative score of items 2–10 of the Tanzanian Poverty Scorecard; Higher score = higher wealth

### Data analysis

Data was analyzed using Stata Statistical Software [43]. Sample characteristics are presented using frequencies and means by gender. All key study variables were assessed for significant correlational relationship with variables using Pearson’s correlations. Hierarchical robust regression models were fitted to examine the association of affective, cognitive, and behavioral empathy on psychological symptom measures (depression, anxiety, and externalizing behaviors). Pubertal development status and sex were included as covariates in Model 1. Model 2 added dimensions of empathy (affective, cognitive, and behavioral). Model 3 added the significant interaction term of pubertal development status and cognitive empathy.

Structural equation modeling (SEM) was completed to estimate paths from pubertal development status to affective, cognitive, and behavioral empathy; and from each empathy subdimension to psychological symptoms. The structural path model is consistent with previously developed conceptual models and theory identifying paths from pubertal development to empathetic capacity and associated relationships with mental health outcomes. The maximum likelihood method of estimation was used to estimate the SEM and was assessed for goodness of fit. The following statistical criteria was used to evaluate model fit: *RMSEA* < 0.06; *CFI* > 0.90, *TLI* > 0.90, and *SRMR* < 0.08 [44].

## Results

### Sample characteristics

Five hundred and seventy-nine (579) adolescents ages 9–12 were included in the analytic sample of this study (Table 1). The sample included 270 boys (46.6%) and 309 girls (53.4%). The mean age of study participants was 10.48 (*SD =* 0.55). 172 (29.7%) of participants were in 3^rd^ grade, 261 (45.1%) in 4^th^ grade and 146 (25.2%) in 5^th^ grade. 345 (66.6%) of participants lived with both parents and 173 (33.4%) did not live with both parents. The average size of the household of study participants was 5.7 (*SD =* 2.6). Study participants reported a mean score of 61.4 (*SD =* 11.6) on the Tanzanian Poverty Scorecard (range 0–71). At the time of data collection, most recent World Bank estimates reported a basic needs poverty line of TZS 49,320 (approximately USD 21) per adult per month [45]. In 2018, the poverty rate in Tanzania was about 26.4%, and about 49% of the population lived below the international poverty line of USD 1.90 per person per day [45]. Covariates that were not found to be associated with empathy dimensions or psychological outcomes were excluded from subsequent analyses.

Several variables included in this study were significantly correlated although the magnitude of correlation was relatively small (Table 2). Affective empathy was positively associated with female gender (*r* = 0.11; *p* < 0.05) and pubertal development (*r* = 0.10; *p* < 0.05). Behavioral empathy was positively correlated with pubertal development (*r* = 0.09; *p* < 0.05); affective empathy (*r* = 0.31; *p* < 0.001) and cognitive empathy (*r* = 0.25; *p* < 0.001). Depression (*r* = 0.09; *p* < 0.05); and anxiety (*r* = 0.17; *p* < 0.001) were positively correlated with cognitive empathy. Externalizing behaviors were negatively associated with behavioral empathy (*r* = -0.18; *p* < 0.001) and positively associated with depression (*r* = 0.51; *p* < 0.001) and anxiety (*r* = 0.43; *p* < 0.001).

**Table 2:** Pearson correlation coefficients for key analytic variables.

Key Variable	1	2	3	4	5	6	7	8	9
1. Sex	-								
2. Age	0.06	-							
3. PDS	0.03	0.01	-						
4. Affective	0.11*	-0.06	0.10*	-					
5. Cognitive	-0.06	0.08	0.01	0.25***	-				
6. Behavioral	0.02	0.04	0.09*	0.31***	0.08*	-			
7. Depression	0.01	0.01	0.07	-0.03	0.09*	-0.15	-		
8. Anxiety	0.01	0.04	0.07	0.05	0.17***	-0.14	0.75	-	
9.Externalizing Behaviors	-0.03	0.03	-0.02	-0.04	-0.03	-0.18***	0.51***	0.43***	-

Note.

*p < 0.05

**p < 0.01

***p < 0.001

Simple linear regressions on empathy dimension were completed by gender, age, and pubertal development stage (1–4) (Table 3). Results indicate that total empathy was higher in PDS stage 3 compared to the reference category of PDS stage 1 (*β* = 0.24; *p* = 0.024). Female gender was associated with higher affective empathy (*β* = 0.21; *p* = 0.011). PDS stage 3 (*β* = 0.24; *p* = 0.024) and PDS stage 4 (*β* = 0.83; *p* = 0.030) were significantly associated with higher affective empathy. PDS stage 4 was associated with higher scores for cognitive empathy (*β* = 0.09; *p* = 0.021).

**Table 3:** Simple linear regressions by sex, age and PDS stage on empathy.

	Affective			Cognitive			Behavioral			Total		
	*β*	*SE*	*p*	*β*	*SE*	*p*	*β*	*SE*	*p*	*β*	*SE*	*p*
Sex	0.21	0.08	0.011*	-0.12	0.08	0.151	0.04	0.08	0.609	0.08	0.08	0.343
Age	-0.11	0.08	0.167	0.14	0.08	0.067	0.07	0.08	0.338	0.01	0.08	0.864
PDS 2	-0.03	0.10	0.779	-0.16	0.10	0.111	0.18	0.10	0.067	-0.02	0.10	0.863
PDS 3	0.24	0.11	0.024*	0.05	0.11	0.645	0.21	0.11	0.052	0.24	0.11	0.024*
PDS 4	0.83	0.38	0.030*	0.09	0.38	0.021*	0.29	0.38	0.446	0.68	0.38	0.074

Note. PDS stage 1 is the reference category

*p < 0.05

**p < 0.01

***p < 0.001

The results of hierarchical regression analyses on depression, anxiety and externalizing behaviors are presented in Table 4. After models were fitted, we examined the variance inflation factor for all regression results to check for multicollinearity. Variance inflation factors (VIF) ranged from 1.02 to 2.79, well below the conventional cut off indicating regression results are robust to multicollinearity of VIF less than or equal to 10. Model 1 included sex, age and PDS; these variables were not significantly associated with depression, anxiety, or externalizing behaviors. Model 2 included sex, age, PDS and affective, cognitive, and behavioral empathy. Mean PDS was associated with higher depressive symptoms (*β* = 1.56; *p* = 0.044), higher anxiety symptoms (*β* = 0.25; *p* = 0.016), and anxiety (*β* = 0.16; *p* < 0.001). In contrast, behavioral empathy was associated with lower depression symptoms (*β* = -0.47; *p* < 0.001) lower anxiety symptoms (*β* = -0.19; *p* < 0.001) and lower reports of externalizing behaviors (*β* = -0.22; *p* < 0.001). Interaction terms between PDS and empathy dimensions and PDS and sex were tested and found to be insignificant. The interaction term between PDS and cognitive empathy was significant and is included in Model 3. Model 3 results demonstrated the interaction between PDS greater than 0 and higher cognitive empathy dimension scores were associated with higher depression symptoms (*β* = 0.28; *p* = 0.020), and PDS and cognitive empathy variables were no longer significant for depression systems. This indicated that the interaction term explained relatively more variance in the outcome after adjusting for PDS and cognitive empathy variables. In Model 3, behavioral empathy remained significant with similar effect size (*β* = -0.46; *p* < 0.001) in association with lower depression, anxiety, and externalizing behaviors.

**Table 4:** Hierarchical multivariable regressions on psychological symptoms.

		Depression ^ŧ^			Anxiety ^ç^			Externalizing Behaviors^Ø^			
Model		*β*	*SE*	*p*	*β*	*SE*	*p*		*β*	*SE*	*p*
M1	Sex	0.06	0.43	0.884	0.01	0.16	0.962		-0.13	0.18	0.474
	Age	0.09	0.39	0.819	0.13	0.15	0.373		0.12	0.16	0.453
	PDS	1.28	0.78	0.102	0.52	0.29	0.078		-0.13	0.32	0.682
M2	Sex	0.17	0.43	0.688	0.04	0.16	0.795		-0.14	0.18	0.436
	Age	0.06	0.39	0.872	0.12	0.14	0.417		0.17	0.16	0.301
	PDS	1.56	0.77	0.044*	0.58	0.29	0.042*		-0.03	0.32	0.932
	Affective	-0.03	0.10	0.780	0.04	0.04	0.262		0.03	0.04	0.430
	Cognitive	0.25	0.10	0.016*	0.16	0.04	0.000***		-0.03	0.04	0.452
	Behavioral	-0.47	0.12	<0.001***	-0.19	0.05	<0.001***		-0.22	0.05	<0.001***
M3	Sex	0.10	0.43	0.820	0.02	0.16	0.877		-0.16	0.18	0.381
	Age	0.09	0.39	0.827	0.12	0.14	0.397		0.17	0.16	0.286
	PDS	-0.13	1.06	0.901	0.21	0.39	0.598		-0.43	0.44	0.335
	Affective	-0.03	0.09	0.743	0.04	0.04	0.274		0.03	0.04	0.446
	Cognitive	0.03	0.14	0.808	0.11	0.05	0.031*		-0.08	0.06	0.151
	Behavioral	-0.46	0.12	<0.001***	-0.19	0.05	<0.001***		-0.22	0.05	<0.001***
	PDS*Cognitive	0.28	0.12	0.020*	0.06	0.04	0.164		0.07	0.05	0.189

Note. SE = Robust standard errors

*p < 0.05

**p < 0.01

***p < 0.001

ŧ *R*^*2*^ = 0.0048 for step 1, *p* = 0.4277; *R*^*2*^ = 0.0395 for step 2, *p* = 0.0001; *R*^*2*^ = 0.0486 for step 3, *p* = 0.0200

ç *R*^*2*^ = 0.0068 for step 1, *p* = 0.2674; *R*^*2*^ = 0.0648 for step 2, *p* < 0.0001; *R*^*2*^ = 0.0680 for step 3, *p* = 0.1635

Ø *R*^*2*^ = 0.0021 for step 1, *p* = 0.7534; *R*^*2*^ = 0.0348 for step 2, *p* = 0.0003; *R*^*2*^ = 0.0377 for step 3, *p* = 0.1889

### Structural Equation Model (SEM)

**Fig 1:**
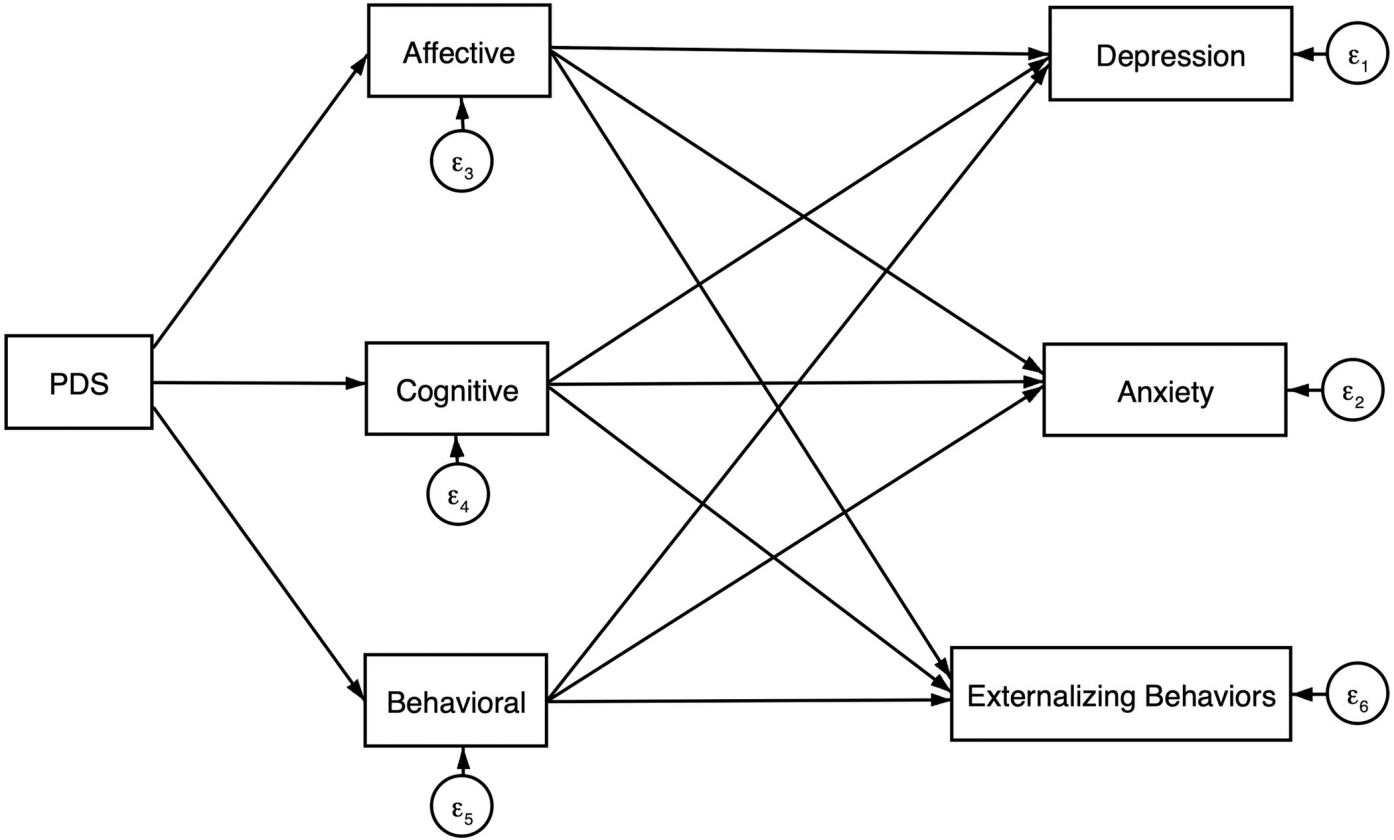
Developmental structural equation model of empathy and psychological symptoms.

**Fig 2:**
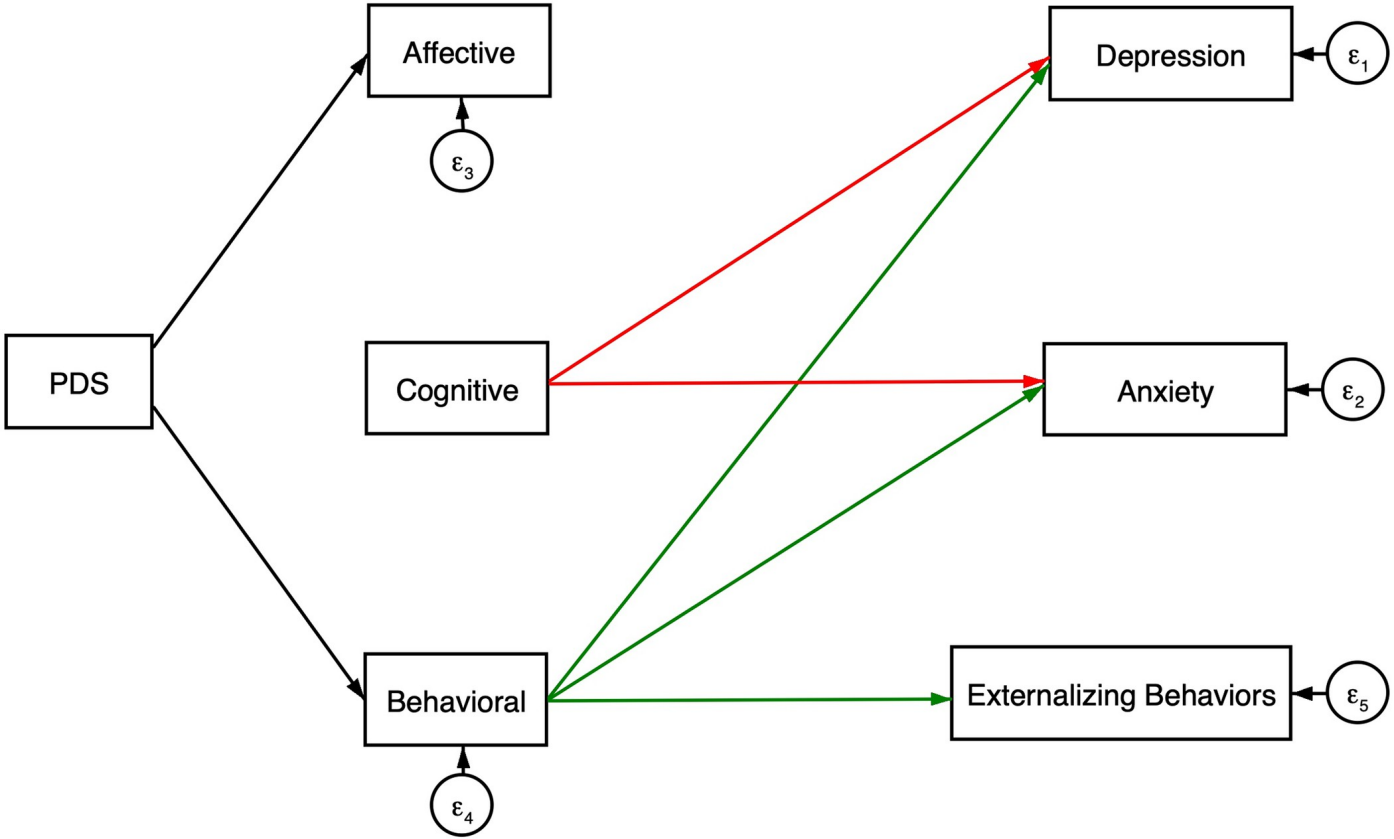
Significant structural paths between pubertal development stage, empathy dimension and psychological symptoms. Note. Green paths are associated with lower psychological symptoms; Red paths are associated with higher psychological symptoms; Black path arrows indicate positive correlation.

Structural paths between pubertal development mean score, affective, cognitive, and behavioral empathy factor scores and depression, anxiety and externalizing symptoms were tested in a structural equation model to evaluate significance of associations by empathy dimension (Fig 1). Goodness of fit indices indicated excellent model fit (*χ*^*2*^, df:3 = 6.656; *p* < 0.001; *CFI* = 0.995; *TLI* = 0.968; *SRMR* = 0.020; *RMSEA* = 0.046; *AIC* = 15404.69; *BIC* = 15535.485). Standardized path coefficients, standard errors, p-values and 95% confidence intervals are listed in Table 5. PDS was associated with higher reported levels of affective (*β* = 0.10; *p* = 0.012) and behavioral (*β* = 0.09; *p* = 0.025) empathy scales, but not cognitive empathy (*β* = 0.01; *p* = 0.880). Higher reported cognitive empathy was associated with more depression (*β* = 0.10; *p* = 0.017) and anxiety symptoms (*β* = 0.18; *p* < 0.001). Behavioral empathy was associated with lower depression (*β* = -0.16; *p* < 0.001), anxiety (*β* = -0.17; *p* < 0.001) and externalizing behaviors (*β* = -0.18; *p* < 0.001). Affective empathy was not associated with any modeled outcomes; however, covariance paths were significant between affective and cognitive (*s* = 0.35; *p* < 0.001), and affective and behavioral (*s* = 0.30; *p* < 0.001) dimensions. The structural equation model is presented in Fig 2 with significant paths only (Fig 2).

**Table 5:** Standardized path coefficients between risk and protective factors and psychological symptoms.

Structural Paths	Coefficient	*SE*	*Z*	*p*>|*z*|	95% Confidence Interval	
**Affective Empathy**						
PDS	0.10	0.04	2.52	0.012*	0.02	0.18
**Cognitive Empathy**						
PDS	0.01	0.04	0.15	0.880	-0.08	0.09
**Behavioral Empathy**						
PDS	0.09	0.04	2.23	0.025*	0.01	0.17
**Depression**						
Affective Empathy	0.00	0.04	-0.08	0.932	-0.09	0.08
Cognitive Empathy	0.10	0.04	2.38	0.017*	0.02	0.18
Behavioral Empathy	-0.16	0.04	-3.68	<0.001***	-0.24	-0.07
**Anxiety**						
Affective Empathy	0.06	0.04	1.27	0.205	-0.03	0.14
Cognitive Empathy	0.18	0.04	4.26	<0.001***	0.09	0.26
Behavioral Empathy	-0.17	0.04	-4.02	<0.001***	-0.25	-0.09
**Externalizing Behaviors**						
Affective Empathy	0.03	0.04	0.61	0.545	-0.06	0.11
Cognitive Empathy	-0.03	0.04	-0.60	0.551	-0.11	0.06
Behavioral Empathy	-0.18	0.04	-4.31	<0.001***	-0.27	-0.10
**Covariance**						
Affective, Cognitive	0.35	0.04	6.54	<0.001***	0.18	0.33
Affective, Behavioral	0.30	0.04	8.05	<0.001***	0.23	0.38
Cognitive, Behavioral	0.08	0.04	1.94	0.052	0.00	0.16
Depression, Anxiety	0.75	0.02	40.79	<0.001***	0.71	0.78
Depression, Externalizing	0.50	0.03	15.90	<0.001***	0.44	0.56
Anxiety Externalizing	0.43	0.03	12.56	<0.001***	0.36	0.49

Note. *χ*^*2*^ = 6.656; df(3); *p* < 0.001; *CFI* = 0.995; *TLI* = 0.968; *SRMR* = 0.020; *RMSEA* = 0.046; *AIC* = 15404.69; *BIC* = 15535.485

## Discussion

The primary aim of this study was to evaluate pubertal development status, dimensional constructs of empathy and associations with psychological symptoms in early adolescents. Cognitive empathy, or the ability to intellectually feel the distress of another person was associated with higher depressive and anxiety symptoms and may be related to co-rumination, a risk factor for internalizing and externalizing problems [46–48]. While co-rumination was not measured in this study, cognitive empathy is plausibly related to co-rumination, the tendency to frequently discuss and rehash problems with peers, because of the capacity to intellectually feel distress of others, without having the skills necessary to intentionally alleviate this distress by effectively comforting others may result in the tendency to co-ruminate [14]. A study on rumination as a transdiagnostic factor in anxiety and depression by McLaughlin & Nolen-Hoeksema, 2011, found that rumination was a more significant mediator in the co-occurrence of symptoms of depression and anxiety in adolescents than adults [49]. McLaughlin & Nolen-Hoeksema also noted no findings of rumination as a mediator of anxiety and later increases in depressive symptoms among adolescents, contrasting other prospective studies [49]. They hypothesized this discrepancy may have resulted from differences in the demographics of the adolescent samples between studies, with their study sampling more economically disadvantaged and minority racial/ethnic groups [49]. These differences highlight the need not only for longitudinal data to examine trajectories of development and change in these relationships, but also the importance of comparing these results across samples of adolescents from diverse cultural and socioeconomic backgrounds to understand how and when cognitive empathy and rumination impact mental health.

After examining sex, age and pubertal development status in relation to empathy dimensions and outcomes, it was determined that PDS, but not age or sex were significantly associated with both empathy dimensions and psychological symptoms in this sample. The failure of age to be significantly associated with empathy and psychological symptoms may be partially attributable to the variation in the age in onset of puberty, particularly between different genders. Sex, while associated with affective empathy, was not associated with the primary outcomes after controlling for PDS and age. While evidence supports gender differences in mental health outcomes, it is likely that the narrow age range of early adolescents included in this sample represents early development, prior to differentiation in mental health outcomes that widen during mid to later adolescence. This finding further underscores the importance of considering pubertal development stage, more than age and gender when assessing empathy and psychological symptoms in very young adolescents. Moreover, in this sample the interaction term between PDS and higher cognitive empathy was significant and indicates the important developmental association with risk for psychopathology.

Behavioral empathy, and the acquisition of skills to practice empathy through intentional action to comfort others was associated with lower depressive, anxiety and externalizing symptoms. Interventions targeting behavioral empathy should consider tailoring approaches and content to specific developmental stages, such as early adolescence, to protect against risk for psychopathology before vulnerability increases during mid to later adolescence [9]. Behavioral empathy and effectively comforting others are skills that require experiential practice to master and younger adolescents may have fewer opportunities than older adolescents or adults to practice these skills. Acquisition of behavioral empathy skills also holds potential to promote wellbeing such as increasing friendship quality, prosocial behaviors and social support systems during a critical developmental period [10–12]. Interventions should consider duration and frequency of interventions targeting empathetic capacity, because researchers have hypothesized that cognitive components of empathy may require longer, sustained interventions than affective components [50]. Research is needed to consider the duration and frequency needed to support behavioral empathy, a newer dimension of empathy that our results indicate are associated with lower internalizing and externalizing symptoms.

Previous research notes that there can be large variations in empathy during development, even within a particular grade level, which can greatly impact the effectiveness of an intervention [50]. To generate developmentally informed intervention approaches it is important to move beyond age as a proxy for development. Use of early screening and developmental assessment tools can improve program planning. Early adolescents ages 10–14, have wide variation in pubertal development stage as well, especially between sexes, with girls transitioning through puberty earlier than boys on average [51, 52]. This further emphasizes the importance of a tailored approach that aligns interventions to developmental stage and promotes appropriate skills and approaches that fit within the zone of proximal development.

More research is needed to evaluate how social emotional learning interventions target or affect affective, behavioral and cognitive empathy dimensions. In addition research should evaluate possible associations between cognitive empathy and co-rumination. Given the promising protective paths from behavioral empathy to depression, anxiety and externalizing behavior, this study suggests that behavioral empathy may be especially important to support during early adolescence.

### Limitations

This study was limited to cross-sectional data. Longitudinal data is needed to identify causal relationships in empathy trajectories across development. Longitudinal research should also consider potential differential impacts of socioeconomic status and cultural factors on the associations between measures such as empathy, pubertal development stage, rumination, and psychological symptoms and mental health. Additionally, the measures of behavioral empathy captured by the “intent to comfort” subscale of the EmQue-CA does not capture actual actions that the young person performed. Further research to assess what kinds of behaviors are appropriate for early adolescents to act on and that provide protection from mental health disorders are important to identify. We recommend additional experimental and longitudinal research to better assess developmental trajectories in causal relationships of empathetic dimensions and psychological outcomes.

## Conclusion

These findings support the importance of examining developmental stage during early adolescence over age in testing predictors of mental health outcomes. Programs targeting empathy should consider the components of the intervention and whether they target affective, cognitive, or behavioral dimensions. To strengthen the translation of research to practice it is important to identify dimensions of empathy as mechanisms of change, and implications for mental wellbeing.

## Supporting information

S1 Dataset Empathy Tanzania year 3 timepoint 1 data. Datafile used for statistical analysis.
(DTA)

## References

[1] Batson CD, Fultz J, Schoenrade PA. Distress and empathy: Two qualitatively distinct vicarious emotions with different motivational consequences. Journal of personality. 1987;55(1):19–39. doi: 10.1111/j.1467-6494.1987.tb00426.x 3572705

[2] Sesso G, Brancati GE, Fantozzi P, Inguaggiato E, Milone A, Masi G. Measures of empathy in children and adolescents: A systematic review of questionnaires. World Journal of Psychiatry. 2021;11(10):876. doi: 10.5498/wjp.v11.i10.876 34733649 PMC8546775

[3] Schonert-Reichl KA, Smith V, Zaidman-Zait A, Hertzman C. Promoting children’s prosocial behaviors in school: Impact of the “Roots of Empathy” program on the social and emotional competence of school-aged children. School Mental Health. 2012;4(1):1–21.

[4] Lawrence EJ, Shaw P, Baker D, Baron-Cohen S, David AS. Measuring empathy: reliability and validity of the Empathy Quotient. Psychological medicine. 2004;34(5):911–20. doi: 10.1017/s0033291703001624 15500311

[5] Van der Graaff J, Carlo G, Crocetti E, Koot HM, Branje S. Prosocial behavior in adolescence: gender differences in development and links with empathy. Journal of youth and adolescence. 2018;47(5):1086–99. doi: 10.1007/s10964-017-0786-1 29185207 PMC5878203

[6] Clark AJ, Butler CM. Empathy: An integral model in clinical social work. Social Work. 2020;65(2):169–77. doi: 10.1093/sw/swaa009 32266410

[7] Eisenberg N, Shea CL, Carlo G, Knight GP. Empathy-related responding and cognition: A “chicken and the egg” dilemma. Handbook of moral behavior and development: Psychology Press; 2014. p. 85–110.

[8] Decety J, Michalska KJ, Akitsuki Y, Lahey BB. Atypical empathic responses in adolescents with aggressive conduct disorder: a functional MRI investigation. Biological psychology. 2009;80(2):203–11. doi: 10.1016/j.biopsycho.2008.09.004 18940230 PMC2819310

[9] Malti T, Noam GG. A developmental approach to the prevention of adolescent’s aggressive behavior and the promotion of resilience. International Journal of Developmental Science. 2009;3(3):235–46.

[10] Chakrabarti B, Baron-Cohen S. Empathizing: neurocognitive developmental mechanisms and individual differences. Progress in brain research. 2006;156:403–17. doi: 10.1016/S0079-6123(06)56022-4 17015093

[11] Knafo A, Zahn-Waxler C, Van Hulle C, Robinson JL, Rhee SH. The developmental origins of a disposition toward empathy: Genetic and environmental contributions. Emotion. 2008;8(6):737. doi: 10.1037/a0014179 19102585

[12] Soenens B, Duriez B, Vansteenkiste M, Goossens L. The intergenerational transmission of empathy-related responding in adolescence: The role of maternal support. Personality and Social Psychology Bulletin. 2007;33(3):299–311. doi: 10.1177/0146167206296300 17312313

[13] Overgaauw S, Rieffe C, Broekhof E, Crone EA, Güroğlu B. Assessing empathy across childhood and adolescence: Validation of the Empathy Questionnaire for Children and Adolescents (EmQue-CA). Frontiers in psychology. 2017;8:870. doi: 10.3389/fpsyg.2017.00870 28611713 PMC5447078

[14] Stone LB, Hankin BL, Gibb BE, Abela JR. Co-rumination predicts the onset of depressive disorders during adolescence. Journal of abnormal psychology. 2011;120(3):752. doi: 10.1037/a0023384 21500877 PMC3140586

[15] MacBeth A, Gumley A. Exploring compassion: A meta-analysis of the association between self-compassion and psychopathology. Clinical psychology review. 2012;32(6):545–52. doi: 10.1016/j.cpr.2012.06.003 22796446

[16] Au AM, Wong AS, Lai MK, Chan CC. Empathy, coping, social support, and mental health in local and migrant adolescents in Beijing. 2011.

[17] Morelli SA, Lieberman MD, Zaki J. The emerging study of positive empathy. Social and Personality Psychology Compass. 2015;9(2):57–68.

[18] Jolliffe D, Farrington DP. Development and validation of the Basic Empathy Scale. Journal of adolescence. 2006;29(4):589–611. doi: 10.1016/j.adolescence.2005.08.010 16198409

[19] Meuwese R, Cillessen AH, Güroğlu B. Friends in high places: A dyadic perspective on peer status as predictor of friendship quality and the mediating role of empathy and prosocial behavior. Social Development. 2017;26(3):503–19.

[20] Okun MA, Shepard SA, Eisenberg N. The relations of emotionality and regulation to dispositional empathy-related responding among volunteers-in-training. Personality and Individual Differences. 2000;28(2):367–82.

[21] Mehrabian A, Epstein N. A measure of emotional empathy. Journal of personality. 1972. doi: 10.1111/j.1467-6494.1972.tb00078.x 4642390

[22] Hudson-Flege MD, Grover HM, Meçe MH, Ramos AK, Thompson MP. Empathy as a moderator of sexual violence perpetration risk factors among college men. Journal of American college health. 2020;68(2):139–47. doi: 10.1080/07448481.2018.1536055 30570441 PMC11102042

[23] Zych I, Ttofi MM, Farrington DP. Empathy and callous–unemotional traits in different bullying roles: A systematic review and meta-analysis. Trauma, Violence, & Abuse. 2019;20(1):3–21. doi: 10.1177/1524838016683456 30803395

[24] Kerr-Gaffney J, Harrison A, Tchanturia K. Cognitive and Affective Empathy in Eating Disorders: A Systematic Review and Meta-Analysis. Frontiers in Psychiatry. 2019;10. doi: 10.3389/fpsyt.2019.00102 30886590 PMC6410675

[25] De Waal FB. Putting the altruism back into altruism: the evolution of empathy. Annu Rev Psychol. 2008;59:279–300. doi: 10.1146/annurev.psych.59.103006.093625 17550343

[26] Milone A, Cerniglia L, Cristofani C, Inguaggiato E, Levantini V, Masi G, et al. Empathy in youths with conduct disorder and callous-unemotional traits. Neural plasticity. 2019;2019. doi: 10.1155/2019/9638973 31097957 PMC6487083

[27] Georgiou G, Kimonis ER, Fanti KA. What do others feel? Cognitive empathy deficits explain the association between callous-unemotional traits and conduct problems among preschool children. European Journal of Developmental Psychology. 2019;16(6):633–53.

[28] Kimonis ER, Frick PJ, Munoz LC, Aucoin KJ. Callous-unemotional traits and the emotional processing of distress cues in detained boys: Testing the moderating role of aggression, exposure to community violence, and histories of abuse. Development and psychopathology. 2008;20(2):569–89. doi: 10.1017/S095457940800028X 18423095

[29] Martin-Key N, Allison G, Fairchild G. Empathic accuracy in female adolescents with conduct disorder and sex differences in the relationship between conduct disorder and empathy. Journal of abnormal child psychology. 2020;48(9):1155–67. doi: 10.1007/s10802-020-00659-y 32488571 PMC7392945

[30] Moul C, Hawes DJ, Dadds MR. Mapping the developmental pathways of child conduct problems through the neurobiology of empathy. Neuroscience & Biobehavioral Reviews. 2018;91:34–50. doi: 10.1016/j.neubiorev.2017.03.016 28377098

[31] Shamay-Tsoory SG, Aharon-Peretz J, Perry D. Two systems for empathy: a double dissociation between emotional and cognitive empathy in inferior frontal gyrus versus ventromedial prefrontal lesions. Brain. 2009;132(3):617–27. doi: 10.1093/brain/awn279 18971202

[32] Preston SD, De Waal FB. Empathy: Its ultimate and proximate bases. Behavioral and brain sciences. 2002;25(1):1–20. 12625087 10.1017/s0140525x02000018

[33] Huang H, Su Y. Peer acceptance among Chinese adolescents: The role of emotional empathy, cognitive empathy and gender. International Journal of Psychology. 2014;49(5):420–4. doi: 10.1002/ijop.12075 24818589

[34] Lin C-T, Chuang S-S. The role of empathy between functional competence diversity and competence acquisition: a case study of interdisciplinary teams. Quality & Quantity. 2018;52(6):2535–56.

[35] Pfeifer JH, Allen NB. Puberty initiates cascading relationships between neurodevelopmental, social, and internalizing processes across adolescence. Biological Psychiatry. 2021;89(2):99–108. doi: 10.1016/j.biopsych.2020.09.002 33334434 PMC8494463

[36] Cherewick M, Lebu S, Su C, Richards L, Njau PF, Dahl RE. Promoting gender equity in very young adolescents: targeting a window of opportunity for social emotional learning and identity development. BMC public health. 2021;21(1):1–18. 34923962 10.1186/s12889-021-12278-3 PMC8684613

[37] Petersen AC, Crockett L, Richards M, Boxer A. A self-report measure of pubertal status: Reliability, validity, and initial norms. Journal of youth and adolescence. 1988;17(2):117–33. doi: 10.1007/BF01537962 24277579

[38] Schmitz KE, Hovell MF, Nichols JF, Irvin VL, Keating K, Simon GM, et al. A validation study of early adolescents’ pubertal self-assessments. The Journal of Early Adolescence. 2004;24(4):357–84.

[39] Cherewick M, Dahl RE, Leiferman J, Hipp E, Schmiege S. Psychometric properties of the empathy questionnaire for children and adolescents (EmQue-CA) in a sample of Tanzanian adolescents. Frontiers in Psychology. 2022;13:981967. 36275217 10.3389/fpsyg.2022.981967 PMC9583906

[40] Betancourt TS, Yang F, Bolton P, Normand SL. Developing an African youth psychosocial assessment: an application of item response theory. International Journal of Methods in Psychiatric Research. 2014;23(2):142–60. doi: 10.1002/mpr.1420 24478113 PMC4098666

[41] Fine SL, Malik A, Guimond M-F, Nemiro A, Temu G, Likindikoki S, et al. Improving mental health in low-resource settings: A feasibility randomized controlled trial of a transdiagnostic psychological intervention among Burundian refugee adolescents and their caregivers. Behaviour research and therapy. 2021;145:103944. doi: 10.1016/j.brat.2021.103944 34392115

[42] Sonderegger R, Rombouts S, Ocen B, McKeever RS. Trauma rehabilitation for war‐affected persons in northern Uganda: A pilot evaluation of the EMPOWER programme. British Journal of Clinical Psychology. 2011;50(3):234–49. doi: 10.1348/014466510X511637 21810104

[43] StataCorp. Stata Statistical Software. 15 ed. College Station, TX, USA: StataCorp LLC; 2017.

[44] Kline RB, Santor DA. [Principles & Practice of Structural Equation Modelling]. Canadian Psychology. 1999;40(4):381.

[45] World Bank. Tanzania Mainland Poverty Assessment. Washington, D.C.; 2019.

[46] Rood L, Roelofs J, Bögels SM, Nolen-Hoeksema S, Schouten E. The influence of emotion-focused rumination and distraction on depressive symptoms in non-clinical youth: A meta-analytic review. Clinical psychology review. 2009;29(7):607–16. doi: 10.1016/j.cpr.2009.07.001 19682781

[47] Verstraeten K, Bijttebier P, Vasey MW, Raes F. Specificity of worry and rumination in the development of anxiety and depressive symptoms in children. British Journal of Clinical Psychology. 2011;50(4):364–78. doi: 10.1348/014466510X532715 22003947

[48] Wadsworth ME, Raviv T, Compas BE, Connor-Smith JK. Parent and adolescent responses to povertyrelated stress: Tests of mediated and moderated coping models. Journal of child and Family Studies. 2005;14(2):283–98.

[49] McLaughlin KA, Nolen-Hoeksema S. Rumination as a transdiagnostic factor in depression and anxiety. Behaviour research and therapy. 2011;49(3):186–93. doi: 10.1016/j.brat.2010.12.006 21238951 PMC3042543

[50] Malti T, Chaparro MP, Zuffianò A, Colasante T. School-based interventions to promote empathy-related responding in children and adolescents: A developmental analysis. Journal of Clinical Child & Adolescent Psychology. 2016;45(6):718–31. doi: 10.1080/15374416.2015.1121822 26890811

[51] Tanner JM. Sequence, tempo, and individual variation in the growth and development of boys and girls aged twelve to sixteen. Daedalus. 1971:907–30.

[52] Greenspan L, Deardorff J. The new puberty: How to navigate early development in today’s girls: Rodale Books; 2014.

