# Supplementary material for: A developmental analysis of dimensions of empathy during early adolescence: Behavioral empathy but not cognitive empathy is associated with lower psychopathology
Source: PLOS Glob Public Health. 2022 Nov 7;2(11):e0001231. doi: 10.1371/journal.pgph.0001231 (PMC10021927; doi:10.1371/journal.pgph.0001231)
Supplement: Attachment — Submitted filename: Response to Reviewers.docx [file pgph.0001231.s002.docx]

22 September 2022

**Response to Reviewers**

PGPH-D-22-01279

**A developmental analysis of dimensions of empathy during early adolescence: Behavioral empathy but not cognitive empathy is associated with lower psychopathology**

Dear Dr. Rakesh Singh,

We are pleased to submit our revised manuscript titled: “**A developmental analysis of dimensions of empathy during early adolescence: Behavioral empathy but not cognitive empathy is associated with lower psychopathology**”, for consideration as a PLOS Global Public Health research article. We would like to sincerely thank you for the opportunity to revise and resubmit our manuscript, and the two anonymous reviewers for their valuable guidance and comments. We have gone through the review comments in detail, and have completed a comprehensive revision of the paper, which addresses and works to accommodate all concerns voiced. We believe that doing so has made the paper much stronger and enhanced its presentation. While we agree with the well-founded criticisms, we are especially encouraged by the following comments:

“The general discussion of the paper on the relevance of generating developmentally informed intervention approaches is very convincing given the results and presents an important contribution to the area of study.”

(Reviewer 1)

“This manuscript covers a topic of interest in the fields of developmental psychology and global mental health...The discussion section contains sound recommendations for future studies given the results of the current study.”

(Reviewer 2)

In preparing the revision of the manuscript, we did not consider the revision requirements as simply compliance with reviewer requests, but rather an opportunity to truly enhance this work and increase its potential to influence the field. For ease of review, we’ve addressed each comment below. We believe we have accommodated all review comments. However, should there be any additional comments we will be happy to accommodate them.

Megan Cherewick, PhD

Department of Community and Behavioral Health

Colorado School of Public Health, University of Colorado Anschutz

13001 East 17^th^ Place

Aurora, CO 80045

Email: [megan.cherewick@cuanschutz.edu](mailto:megan.cherewick@csueastbay.edu)

Journal Requirements

1. Please review your reference list to ensure that it is complete and correct. If you have cited papers that have been retracted, please include the rationale for doing so in the manuscript text, or remove these references and replace them with relevant current references. Any changes to the reference list should be mentioned in the rebuttal letter that accompanies your revised manuscript. If you need to cite a retracted article, indicate the article’s retracted status in the References list and also include a citation and full reference for the retraction notice.

**Thank you for this suggestion. We have passed our references list through the scite.ai reference check software and have found no articles with retracted status in our submission.**

2. Please amend your detailed Financial Disclosure statement. This is published with the article. It must therefore be completed in full sentences and contain the exact wording you wish to be published.
   a. State what role the funders took in the study. If the funders had no role in your study, please state: “The funders had no role in study design, data collection and analysis, decision to publish, or preparation of the manuscript.”

**Thank you for this suggestion. We have amended our Financial Disclosure statement to the following:**

**This publication is based on research funded by the Bill and Melinda Gates Foundation (Grant number: OPP1158584). The funders had no role in study design, data collection and analysis, decision to publish, or preparation of the manuscript. Findings and conclusions contained within are those of the authors and do not reflect positions or policies of the Bill and Melinda Gates Foundation.**

3. We have noticed that you have uploaded Supporting Information files, but you have not included a list of legends. Please add a full list of legends for your Supporting Information files after the references list.

**Thank you for the opportunity to clarify this. The attached Supporting Information document is the article Cherewick, 2022 referenced in line 173, which has now been accepted for publication by Frontiers in Psychology but has not yet been published. The data file of the Supporting Information is the data used for the analyses in this manuscript.**

Reviewer 1

Introduction

1. In lines 72-73 about studies showing association of empathy and prosocial behavior and externalizing problems, it would be interesting to see a slightly more detailed explanation as to what kind of empathy and in what population the results were found, so that the potential of the presented paper is clearer (do we need to understand how it works in a different population such as young adolescents in Tanzania, etc.)

**Thank you for this suggestion, we have modified lines 104-110 of the Introduction to make the specific relationships between empathy dimensions and behaviors clearer. For example, specifying cognitive empathy’s relationship with higher friendship quality.**

2. In lines 104-107 on the brain structure related to empathy, it could help to explain it in lay language so that the non-expert audience can understand easily.

**Thank you, we have modified the language in lines 136-138 to use more lay language to describe the relationship between brain structure and empathy.**

Materials and Methods

1. In lines 135-136 the protocol of the parent study Discover Learning is mentioned and it's indicated that it's provided elsewhere, but it would be interesting to include at least a brief explanation of what it entails and why it is used.

**We appreciate this insight. We have added a brief description of the study on lines 362-364 in addition to providing the reference to the full study protocol.**

2. It's not clear why only verbal assent from adolescents was required, they could also provide written assent unless explained why (culture, literacy level, etc.)

**Thank you for bringing this confusion to our attention. We have described the use of verbal assent on lines 374-376, clarifying that literacy was limited and verbal assent is considered typical in this context.**

3. It is mentioned that each measurement scale was selected based on previous use in low- and middle-income countries with adolescent populations and that items were adapted to reflect the study context and age of participants. Howev, it is not clear what use they gave them in other studies and how the adaptation was done for this study, e.g. it's not explained how the PDS questionnaire was validated in Tanzania, and for AYPA the development scale is mentioned but not for the target population of this study.

**We appreciate this suggestion. In the Pubertal Development Scale section beginning on line 389 we have included a reference to a validation study with the correlations utilized. We have also added sample questions that we believe clarify the scale measures and appropriateness for use with this sample population. For the AYPA scale, we have included references to studies that have previously used the AYPA scale with Tanzanian adolescents with minor adaptations on line 493. For the Empathy scale, we have provided reference to a validation study with Tanzanian adolescents that was recently accepted for publication on line 477.**

4. The poverty profile of the participants is mentioned but not really analyzed.

**Thank you for noting this. We chose to include measures of poverty in order to more clearly demonstrate the low-resource context that the sample population is representative of. We have added clarification on lines 720-721 to indicate that not all demographic measures described are included in the analysis, and rather serve to provide more context to the population being studied.**

Results

1. It looks like something was incorrectly written in line 240, it states: "with cognitive empathy anxiety", probably "anxiety" was incorrectly included.

**Thank you for noting this typographical error, it has been corrected on line 642.**

2. The robustness of the multivariable regression is not clearly explained, if some tests were performed, it would be relevant to show them. For example, if the different empathy dimensions are correlated, or age and PDS are correlated, wouldn't this be presenting problems of multicollinearity?

**Thank you for this comment. We have added to the discussion of the regression analyses to include checking for multicollinearity using the variance inflation factor, and the results of those assessments on lines 657-660.**

Discussion

1. The writing in lines 304-306 seems repetitive.

**Thank you, we have modified the first paragraph of the Discussion for clarity and conciseness.**

2. The studies of rumination and co-rumination suddenly appear here, but they were never mentioned as a framework or basis for the analysis in the introduction. It would be clearer if the concept is introduced in the introduction so that it makes more sense to bring it up in the discussion and analysis of results.

**We appreciate this suggestion and have added to the Introduction in lines 110-113 to introduce the relationship between co-rumination and depression and set the stage for its relevance in the Discussion.**

3. In line 327 is mentioned that an objective was to test sex (in addition to age and PDS) in relation to empathy dimensions and outcomes, but the sex variable is then never analyzed. If there were no relevant results, it would be worth mentioning it.

**Thank you for bringing this to our attention. In lines 823-834 in the Discussion, we added language describing that there was not significant association with empathy or psychological outcomes in this study. We also added to the Results in line 720-721 that indicates that covariates like sex, that were not found to be associated were excluded from the subsequent analyses.**

4. Lines 351-352: what about behavioral components? The previous consideration was on behavior but it’s not mentioned here for the intervention duration.

**Thank you for noting this. We have restructured and added more to the Discussion, which we believe creates a clearer picture of each empathy domain overall, and better addresses considerations with behavioral components. We have addressed this specific point of omission regarding behavioral empathy in lines 847-849 suggesting future research consider duration and frequency needed to support this dimension of empathy.**

5. The general discussion of the paper on the relevance of generating developmentally informed intervention approaches is very convincing given the results and presents an important contribution to the area of study.

**Thank you for this positive feedback, as well as the valuable constructive criticism that has helped us improve the clarity and quality of our submission.**

Limitations

1. The limitations are clear and well thought to move forward in the field.

**Thank you for this positive feedback, which is both encouraging and helpful for guiding future work.**

Reviewer 2

1. This manuscript covers a topic of interest in the fields of developmental psychology and global mental health.

**Thank you for this positive feedback and the valuable constructive criticisms that help improve our contribution to the field.**

2. The authors of the manuscript may want to provide more detail on their definition of externalizing behaviors so readers have a better sense of the how cognitive and behavioral empathy relate to certain behaviors.

**Thank you for this suggestion. To clarify what kind of behaviors are encompassed by externalizing behaviors, we have added some examples to line 105.**

3. The alpha level and a sample question from the Pubertal Development Scale should also be reported.

**We appreciate this feedback and have included the alpha level and several examples for the PDS for both boys and girls in lines 341-344.**

4. The discussion section contains sound recommendations for future studies given the results of the current study.

**We are grateful for the positive feedback and your assistance in improving this submission.**

5. It is recommended that the authors review the manuscript for minor typos to ensure that ideas are communicated clearly.

**Thank you, we have reviewed the manuscript over for typos and grammatical errors.**
